# Cholesterol in human atherosclerotic plaque is a marker for underlying disease state and plaque vulnerability

**DOI:** 10.1186/1476-511X-9-61

**Published:** 2010-06-11

**Authors:** Zhu Chen, Marina Ichetovkin, Marc Kurtz, Emanuel Zycband, Douglas Kawka, John Woods, Xuanmin He, Andrew S Plump, Eric Hailman

**Affiliations:** 1 Cardiovascular Diseases, Merck Research Laboratories, 126 E. Lincoln Ave., Rahway, NJ 07065, USA; 2 Target Validation, Merck Research Laboratories, 126 E. Lincoln Ave., Rahway, NJ 07065, USA; 3 FoxHollow Technologies Division, EV3 Inc., 740 Bay Road, Redwood City, CA 94063, USA

## Abstract

**Background:**

Cholesterol deposition in arterial wall drives atherosclerosis. The key goal of this study was to examine the relationship between plaque cholesterol content and patient characteristics that typically associate with disease state and lesion vulnerability. Quantitative assays for free cholesterol, cholesteryl ester, triglyceride, and protein markers in atherosclerotic plaque were established and applied to plaque samples from multiple patients and arterial beds (Carotid and peripheral arteries; 98 lesions in total).

**Results:**

We observed a lower cholesterol level in restenotic than primary peripheral plaque. We observed a trend toward a higher level in symptomatic than asymptomatic carotid plaque. Peripheral plaque from a group of well-managed diabetic patients displayed a weak trend of more free cholesterol deposition than plaque from non-diabetic patients. Plaque triglyceride content exhibited less difference in the same comparisons. We also measured cholesterol in multiple segments within one carotid plaque sample, and found that cholesterol content positively correlated with markers of plaque vulnerability, and negatively correlated with stability markers.

**Conclusions:**

Our results offer important biological validation of cholesterol as a key lipid marker for plaque severity. Results also suggest cholesterol is a more sensitive plaque marker than routine histological staining for neutral lipids.

## Background

Atherosclerosis is a chronic disease characterized by lipid deposition and inflammation in the arterial wall[1]. Cholesterol is the major lipid species in atherosclerotic lesions and accumulates in both unesterified and esterified forms[2]. Early studies characterizing localization and abundance of cholesterol in association with lesion morphology and severity have illuminated the prominent role of cholesterol in athero-progression. Rapp et al. observed that an increased percentage of free cholesterol (as a proportion of total cholesterol) was associated with evolution of the atherosclerotic process[3]. Kruth further demonstrated that free and esterified cholesterol accumulated within many diverse and distinct structures in lesions, and extracellular free cholesterol-enriched particles constituted a significant portion of accumulated cholesterol[4]. Klemp et al. found that lesion cores showed an increase in the percentage of free cholesterol, whereas lesion caps were more enriched in cholesteryl ester[5]. These studies in aggregate suggest that deposition of free and esterified cholesterol has a critical physiological impact on athero-progression.

A large body of evidence supporting cholesterol's central role in atherosclerosis was generated using histochemical analysis with lipid-soluble Sudan dyes such as Oil Red O (ORO). These dyes stain neutral lipids including cholesteryl ester and triglyceride, but they do not stain free cholesterol[6]. Nonetheless, such stains in human plaque have demonstrated that lipid enrichment is usually associated with higher degrees of inflammation and characteristics of plaque vulnerability[7].

In addition to being a key driver for atherosclerosis, cholesterol is dynamically modulated in lesion regression or stabilization. A robust reduction in free and esterified cholesterol in response to treatment with certain agents has been observed in animal studies[8-10]. In clinical studies, although quantitation of lesion cholesterol has not been utilized as study outcome, histological analyses have demonstrated that certain therapies, such as statins, can result in reduction in lipid-rich region in lesions[11].

In our effort of using plaque as a quantitative platform for studying effect of novel therapeutic entities in humans, we sought to explore the relationship between plaque cholesterol content and patient characteristics that have been implicated in lesion pathogenesis. We analyzed free and esterified cholesterol levels, along with other lipid and protein markers, in lesions from three stratified patient cohorts. Correlations between cholesterol levels and patient characteristics constitute key results in this report. We also provide an analysis of cholesterol content in different segments within one plaque as additional validation of our approach.

## Methods

### Human atherosclerotic lesions

For the restenotic vs. primary plaque comparison, 11 restenotic plaques and 36 primary plaques were obtained through atherectomy with the Silverhawk apparatus (EV3, Inc.) from lower extremity of patients. Samples were flash frozen and stored at -80°C till processing. For the diabetic (DM) vs. non-diabetic (non-DM) comparison, peripheral plaques from 14 DM patients and 16 non-DM patients were obtained through the Silverhawk apparatus. Diabetic status was based on patient report at the time of atherectomy. Additional patient characteristics are described in Table 1. Samples were collected into RNAlater solution (Qiagen) and stored at -20°C till processing. For the symptomatic vs. asymptomatic comparison, plaques from 8 symptomatic patients (75 ± 7 years of age, 7 males) and 12 asymptomatic patients (71 ± 13 years of age, 6 males) were obtained through carotid endarterectomy. The plaques were categorized by the patient's physician as either symptomatic or asymptomatic according to patient's history and clinical examination. Samples were collected into RNAlater and stored at -20°C till processing. For the sub-plaque analysis, one plaque was obtained from a carotid endarterectomy patient at Ellis Hospital (Schenectady NY), embedded and stored frozen in Optimal Cutting temperature (OCT) solution until processing. Institutional Review Board (IRB) approval was obtained and patients gave informed consent for use of their plaque tissues in studies of tissue biomarkers.

**Table 1:** Patient Characteristics of the Diabetic (DM) and Non-diabetic (non-DM) Groups

Patient data	Non-DM	DM	P value
Male gender (total)	10 (16)	7 (14)	N/A
smoking (total)	12 (16)	10 (14)	N/A
age, years	73+/-10	72+/-13	>0.1
HbA1C	5.8+/-0.7	7.1+/-1.5	0.003
Total Cholesterol, mg/dL	189+/-58	154+/-26	0.05
HDL, mg/dL	52+/-21	39+/-13	0.06
LDL, mg/dL	104+/-35	86+/-23	>0.1
Triglycerides, mg/dL	196+/-150	145+/-85	>0.1

### Protein extraction and measurement

RNAlater-stored plaques were removed from RNAlater and powderized by TissueLyser (Qiagen) at -80°C before processing for protein. Flash frozen plaques were pulverized by Covaris Tissue CryoPrep system (Covaris, Inc.) at -80°C before processing for protein. Tissue slices from the OCT-embedded carotid plaque were also pulverized. Protein in the pulverized or powderized samples were extracted with PBS + 1%CHAPS in the Covaris E210 tissue extraction system and stored at -80°C until assay. Pellets were stored at -80°C until processing for lipids. CD68 was measured using an immunoassay developed for human CD68. The calibrator for the assay was a lysate of the macrophage cell line THP-1, hence the results of CD68 measurement are expressed as μg of THP-1 lysate. ICAM-1 levels were determined by Rules Based Medicine Human MAP multi-analyte analysis. Lumican and Smooth Muscle Myosin Heavy Chain (SMMHC) levels were determined by Western blotting, with the goat anti-Lumican (R&D Systems) and mouse anti-SMMHC (Dako) used as primary antibodies. Cathepsin B was measured by an immunoassay from R&D Systems.

### Lipid extraction and measurement

Pellets resulted from protein extraction were subjected to lipid extraction by the Folch method[12]. For cholesterol measurement, lipid extracts were spotted onto 96-well plates, dried, and resolubilized in ethanol. To measure free cholesterol, samples were incubated for 1 hour at 37°C in a reaction mixture that includes cholesterol oxidase, peroxidase, and *p*-hydroxyphenylacetic acid, with the fluorescent product measured in a Tecan GENios Pro. Cholesterol esterase (Calbiochem) was included in separate reactions for measuring total cholesterol. Cholesteryl ester content was derived by subtracting free cholesterol from total cholesterol. For triglyceride measurement, lipid extracts were spotted onto 96-well plates, dried, and resolubilized in 4% Triton X-100. Triglyceride assay reagent (Roche) was then added to each well to allow reaction at room temperature for 10 minutes, with the colorimetric product measured in a Spectra Max 250 (Molecular Devices).

### Histology and immunohistochemistry

10 μm tissue sections were generated from the OCT-embedded plaque by Cryostat. Hemotoxylin & eosin (H&E) staining was performed by routine methods. Mouse anti-CD68 (Neomarkers, Ab-3) and mouse anti-SMMHC (Neomarkers, SMMS-1) were used for immunohistochemistry for CD68 and SMMHC, respectively.

### Data Analyses

For all whole plaque samples, the analytes' values were normalized to tissue weight. Cholesterol and triglyceride were expressed as nmol/mg tissue, and Lumican was expressed in units of AU (arbitrary unit) per mg tissue. For the OCT-embedded plaque slices, the analytes' values were normalized to total protein. Cholesterol and triglyceride were expressed as nmol/mg protein; ICAM-1, CD68, SMMHC were expressed as ng/mg protein, μg THP-1/mg protein, and AU/mg protein, respectively. T-tests were generated using Microsoft Excel's functions. For comparison between patient groups, natural logarithm transformation was applied to the normalized analytes' levels to meet the normality assumption for statistical analyses.

## Results

### Restenotic plaque had lower cholesterol content than primary plaque

**Figure 1:**
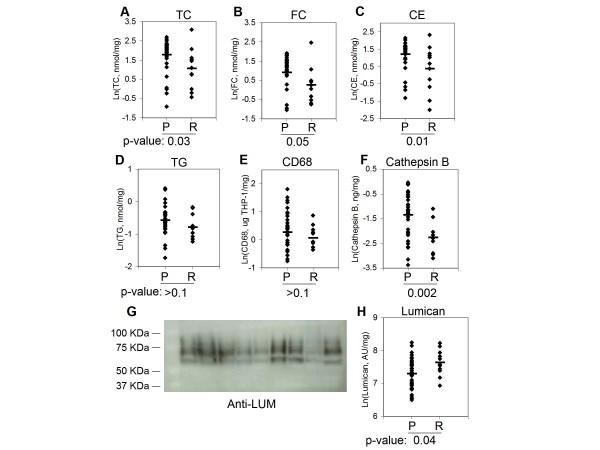
**47 peripheral plaque samples were sequentially extracted for protein and lipid**. TC (**A**), FC (**B**), CE (**C**), TG (D), CD68 (E), Cathepsin B (F), and Lumican (G, H) levels in the plaque were determined and compared between restenotic (R) and primary (P) plaques. Bar represents mean.

Several studies have indicated restenotic plaques are frequently associated with lower prevalence of large lipid core and inflammation compared with primary lesions[13,14], although specific comparison of cholesterol content has not been reported. We measured cholesterol content in 11 restenotic and 36 primary peripheral plaques, and observed significantly less total cholesterol (TC) (Fig. 1A), free cholesterol (FC) (Fig. 1B), and cholesteryl ester (CE) (Fig. 1C) in the restenotic group. Triglyceride (TG) trended toward a lower level in the restenotic group without reaching statistical significance (Fig.1D). CD68, a marker for macrophage content, was not distinguishable between the two groups (Fig. 1E), whereas Cathepsin B, a protease that has been linked to plaque inflammation and vulnerability[15], was significantly lower in the restenotic group (Fig. 1F). These results suggest that although macrophage content was similar between the two groups, macrophages in restenotic plaque may display less inflammatory and proteolytic activities.

Since it is known that restenotic plaque has proteoglycan-enriched extracellular matrix[16], we were curious about a small soluble proteoglycan, Lumican, in this study. Lumican was previously characterized as dynamically present in various stages of lesion progression and potentially involved in smooth muscle proliferation[17,18]. Our analysis revealed Lumican was highly abundant in plaque samples, with its SDS-PAGE migration pattern similar to previously observed[17] (Fig. 1G); restenotic lesions had significantly more Lumican than primary lesions (Fig. 1H), suggesting that Lumican may indeed be involved in the smooth muscle cell proliferation process underlying restenosis.

### Symptomatic carotid plaque trended toward more cholesterol deposition than asymptomatic plaque

**Figure 2:**
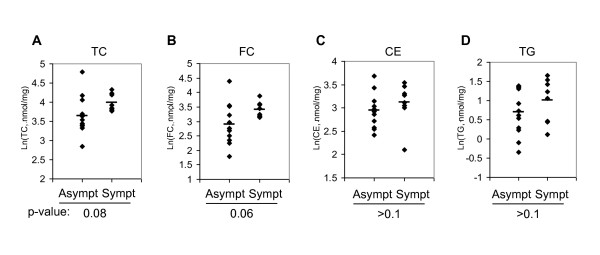
**20 carotid plaque samples were sequentially extracted for protein and lipid**. TC (**A**), FC (**B**), CE (**C**), and TG (D) levels in the plaque were determined and compared between the asymptomatic and symptomatic groups. Bar represents mean.

We measured cholesterol, triglyceride, and inflammatory protein markers in carotid plaques from 8 symptomatic patients (those with a history of neurological symptoms attributable to carotid atherosclerosis) and 12 asymptomatic patients. TC (Fig. 2A) and FC (Fig. 2B) trended toward a higher level in symptomatic plaque, with their p-values approaching statistical significance. CE (Fig. 2C) and TG (Fig. 2D) exhibited a similar, albeit weaker, trend. The fact that FC was more distinguishable than CE between the two groups is consistent with the understanding that accumulated FC is highly toxic and contributes to lesion progression and instability[3]. Our results also demonstrated that among the major lipid species in plaque, cholesterol had a stronger correlation with cerebrovascular events than triglyceride, which is consistent with the notion that cholesterol is the primary driver for atherosclerosis.

### Cholesterol content in diabetic vs. non-diabetic peripheral plaques

**Figure 3:**
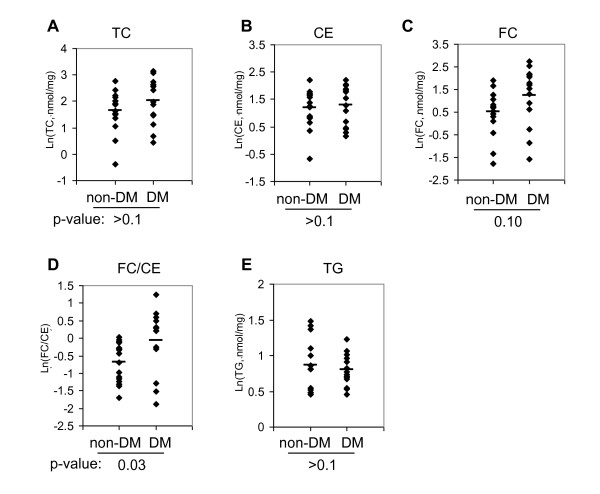
**30 peripheral plaque samples were sequentially extracted for protein and lipid**. TC (**A**), CE (**B**), FC (**C**), FC/CE (D), and TG (E) levels were determined and compared between the non-diabetic (non-DM) and diabetic (DM) groups. Bar represents mean.

Diabetic patients are at a high risk for cardiovascular events compared with non-diabetics with similar atherosclerotic burden[19], and several studies have found features of plaque vulnerability in diabetic patients, including increased lipid and macrophage content and decreased collagen content[20,21]. We examined cholesterol content in peripheral plaque samples from 14 diabetic and 16 non-diabetic patients. TC (Fig. 3A) and CE (Fig. 3B) were barely distinguishable between the two groups. FC displayed a weak trend toward higher deposition in the diabetic group (Fig. 3C). FC to CE ratio was significantly higher in the diabetic group (Fig. 3D), suggesting those lesions may be more advanced. TG content was indistinguishable between the two groups (Fig. 3E). It should be noted that in this cohort of apparently well-managed patients, lipid profiles of the diabetic group were not typical of diabetic dyslipidaemia, with their serum cholesterol and triglyceride levels lower than the non-diabetic group (Table 1).

### Cholesterol analysis in multiple segments within one carotid plaque

**Figure 4:**
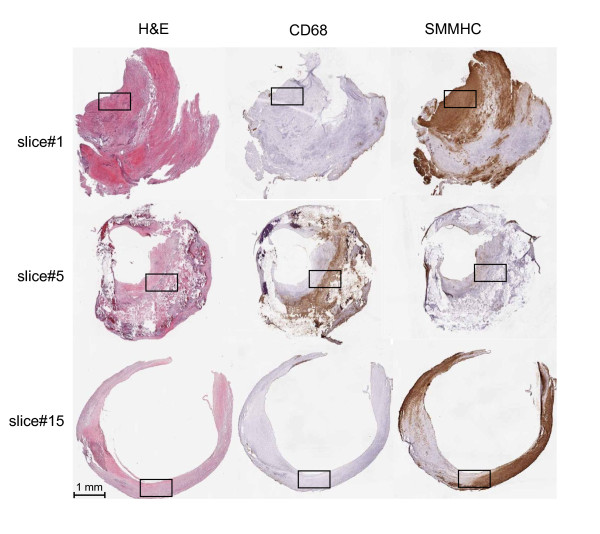
**A carotid plaque was alternately sectioned to allow histological, protein, and lipid analyses**. Macroscopic views of H&E, CD68, and SMMHC stainings for selected slices are shown.

**Figure 5:**
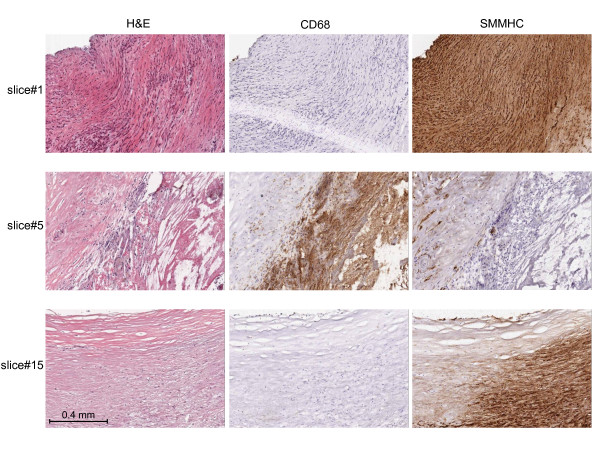
**High-power view of the insets in Fig. 4**.

**Figure 6:**
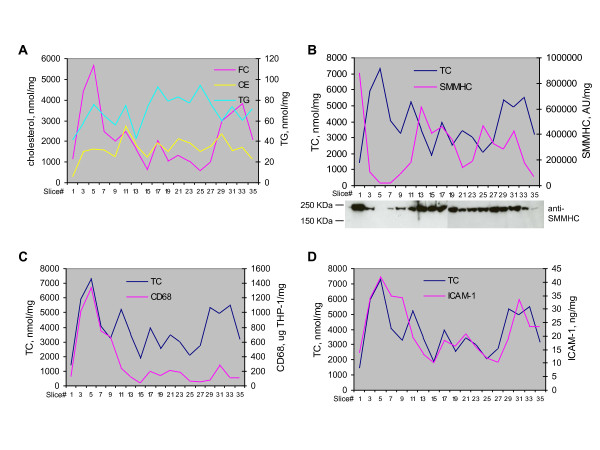
**(A) TG and cholesterol levels over the segments**. (B-D) SMMHC, CD68, and ICAM-1 levels in comparison to TC.

Atherosclerotic lesions are heterogeneous, and lipid deposits within a plaque can differ markedly, accompanying distinct local morphological characteristics[5]. We therefore sought to examine the relationship between cholesterol levels and morphological and molecular features in multiple segments (slices) within one plaque specimen. An OCT-embedded carotid plaque was serially cut into cross-sections on a cryostat, with 300 μm slices used for protein and cholesterol measurement, and adjacent thin (10 μm) sections used for histological analyses. Macroscopic and microscopic views of H&E, CD68, and SMMHC stainings in sections adjacent to and representing slice#1, 5, and 15, were provided in Fig. 4 and Fig. 5, respectively, for illustration of their key morphological features. Slice#1 was highly stenosed with intense SMMHC staining and low CD68 staining, indicating it was primarily a smooth muscle cell-rich, fibrous segment. Slice#5 had extensive calcification and strong CD68 staining in a large lipid/necrotic core with an overlaying fibrous cap. These features, along with its low SMMHC positivity, suggest it is a vulnerable segment within the plaque. Slice#15, in comparison, was at the thickened intima stage with slight calcification, low CD68 staining, and high SMMHC staining. When we examined lipid distribution over the segments (Fig. 6A), we observed that CE and TG had similar distribution patterns; FC overall tracked with CE except than in the lipid core (slice#5), where cholesterol clefts were readily discernible (Fig. 5). In examining the relationships between cholesterol and protein markers, we found distribution pattern of TC was opposite to a plaque stability marker, SMMHC (Fig. 6B); TC distribution was similar to markers of vulnerability, CD68 (Fig. 6C) and ICAM-1 (Fig. 6D). A second carotid plaque that lacked a prominent lipid core displayed similar, albeit less striking correlations, between cholesterol and inflammatory markers (not shown). Taken together, our observations demonstrate that cholesterol distribution pattern fit with its understood physiological impact and local morphological characteristics.

### Cholesterol content in carotid plaque vs. peripheral plaque

**Figure 7:**
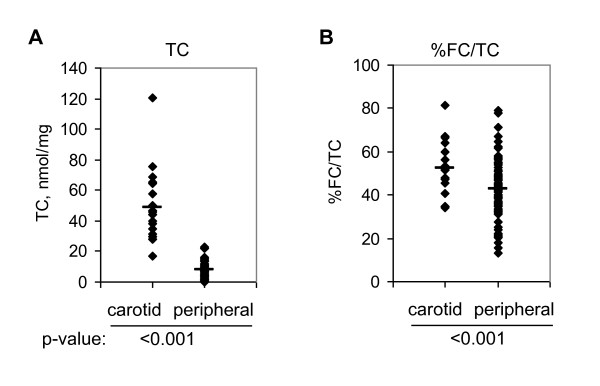
**Cholesterol deposition and FC enrichment in carotid vs. peripheral arteries**. TC content and percentage of FC in TC (%FC/TC) are shown in A and B, respectively. Bar represents mean.

In analyzing data from the above described restenotic vs. primary plaque study, symptomatic vs. asymptomatic plaque study, and diabetic vs. non-diabetic plaque study, we also found carotid plaques had significantly more TC (Fig.7A) and higher percentage of FC than peripheral plaques (Fig.7B).

## Discussion

In this report we made novel observations about human plaque regarding the relationship between plaque cholesterol content and a number of clinical parameters that typically associate with plaque instability. Our findings confirmed and extended the established understanding on free and esterified cholesterol in plaque, validated our approach, and solidified our understanding of atherosclerosis.

In interpreting our results we need to keep in mind that histopathological features of plaque are modifiable by multiple additional clinical parameters and risk factors. For example, a large carotid plaque study by Rothwell and colleagues showed that key lesion characteristics are highly influenced by nature and timing of ischemic events[22], and relationship between lesion characteristics and diabetes displayed a temporal trend as well[23]. Histological features of restenotic plaque are also highly dynamic and dependent on recurrence interval and clinical presentation, with late restenotic lesion starting to resemble primary lesion[14]. It is therefore unsurprising that sample sizes for such binary comparisons are usually too small and results need to be taken with recognition of caveats with such a validation approach. Nonetheless, our observations are consistent with a larger body of literature on plaque inflammation and lipid deposition in relation to neurological symptoms, diabetes, and restenosis.

Our observation that carotid plaques had significantly more TC and higher percentage of FC than peripheral plaques is consistent with previous findings that carotid arteries have a higher prevalence of foam cell lesions and lipid core plaques than peripheral arteries[24]. Our intra-plaque analysis of cholesterol content in comparison with protein markers is also consistent with the understanding of role of cholesterol in plaque vulnerability.

Our specific analyses of FC and CE in the symptomatic/asymptomatic comparison and DM/non-DM comparison suggest FC is a more sensitive marker of plaque severity than CE. From the perspective of drug effect, in animal studies, reduction in CE by therapeutic entities appeared to always exceed reduction in FC[8-10]. Although such comparisons in human lesions are yet to be reported, it is plausible that we will observe the same pattern in human drug trials. Main reason for this expectation is that we believe the predominant mechanism of cholesterol reduction in lesion is cholesterol efflux from CE-enriched cells and a high percentage of FC in advanced human lesions exists in the extracellular space in inaccessible forms. Distinguishing CE from FC is therefore a highly desirable capability of our platform. Animal studies also suggested reduction in CE by drug treatment could exceed reduction in triglyceride[8]. Since both CE and triglyceride stain positive with ORO, our results together with available literature evidence suggest specific quantitation of FC and CE may allow us better sensitivity in gauging plaque severity and drug effect than neutral lipids staining with ORO.

Our cholesterol analyses across different arterial beds and patient groups have also added a value to our platform-building effort in that they provided a new dimension in assessing protein and RNA biomarkers. Indeed, through plaque splitting, which allows parallel measurement of cholesterol, protein, and RNA markers within one tissue sample (manuscript submitted), we have identified a host of protein markers and gene expression signatures that correlate with cholesterol deposition (not shown). Further analysis of these findings will not only validate our approach but also yield new insight on the complex plaque biology. Our lipid extraction and cholesterol measurements also opened the door to establishing and evaluating additional plaque lipid components that are potentially implicated in lesion progression. Finally, our results could also serve as an important benchmark for assessing various newly-emerged imaging modalities that bear the promise of quantitating lipid content in plaque.

## Conclusions

We have integrated cholesterol measurement into our human plaque analysis as a potential platform for reading drug effect. Our studies, to our best knowledge, mark the first effort of correlating plaque cholesterol content, including free and esterified cholesterol, with multiple patient characteristics. Our findings demonstrate that specific and quantitative analysis of cholesterol serves as an excellent marker for plaque vulnerability and potentially a very suitable endpoint for measuring drug effect. Clinical studies in which we will measure a comprehensive endpoint that includes plaque cholesterol, protein, RNA, and imaging parameters in response to drug treatment are under way. Results also support the notion that deposition of cholesterol, in particular free cholesterol, in human atherosclerotic plaque, is a marker for underlying disease state and lesion progression.

## Competing interests

ZC, MI, MK, EZ, DK, JW, AP and EH are current or former employees and/or hold stocks or stock options of Merck & Co. at the time the manuscript was prepared. XH is a former employee and hold stock options of EV3 Inc. at the time of manuscript preparation.

## Authors' contributions

ZC and EH designed the studies, participated in all data interpretation, and drafted the manuscript. AP critically reviewed the manuscript. ZC and MI carried out lipid analysis and data interpretation. ZC and MK carried out protein analysis and data interpretation. EZ, DK, JW, and XH carried out histological analysis and data interpretation. XH coordinated sample acquisition. All authors have read and approved the final manuscript.
